# Lack of efficacy of oral N-acetylcysteine ​​in the treatment of facial melasma in women: a randomized, double-blind, placebo-controlled clinical trial^☆^

**DOI:** 10.1016/j.abd.2023.10.005

**Published:** 2024-08-06

**Authors:** Melissa de Almeida Corrêa Alfredo, Ingrid Rocha Meireles Holanda, Daniel Pinho Cassiano, Ana Cláudia Cavalcante Espósito, Paula Basso Lima, Hélio Amante Miot

**Affiliations:** a Department of Infectology, Dermatology, Imaging Diagnosis and Radiotherapy, Faculty of Medicine, Universidade Estadual Paulista, Botucatu, SP, Brazil; b Department of Dermatology, Universidade Federal de São Paulo, São Paulo, SP, Brazil; c Department of Medicine, Universidade do Oeste Paulista, Unoeste, Presidente Prudente, SP, Brazil

Dear Editor,

Melasma is an acquired chronic dyschromia, more prevalent in menopausal women and characterized by cutaneous hypermelanosis in sun-exposed areas, especially the face. It can be triggered by photo exposure, pregnancy, hormonal changes and medications, but its pathogenesis is not completely understood, reflected in the frequent recurrence after treatment.^1^, ^2^

Oxidative stress, caused by external factors such as exposure to solar radiation, sleep disorders, pollution, skin inflammation and emotional stress, can induce and perpetuate melanogenesis regardless of ultraviolet irradiation. Moreover, eumelanogenesis is an intracellular oxidative process.^1^ Serum markers of oxidative stress are elevated in patients with melasma, with a strong correlation observed between plasma glutathione peroxidase levels and severity of melasma.^1^, ^3^

In recent years, oral and topical antioxidants have shown to be effective in melasma treatment; however, no clinical trials have been conducted with N-acetylcysteine, which constitutes a “thiol” compound and acts as a donor of L-cysteine, leading to replacement and increased levels of intracellular glutathione.^2^, ^4^, ^5^, ^6^ The objective of this study was to evaluate the effectiveness of using oral N-acetylcysteine ​​for eight weeks in the treatment of facial melasma in adult women.

Between April and July 2022, a randomized, parallel, multicenter (Inst1, Inst2, Inst3), double-blind, placebo-controlled clinical trial was conducted on 50 women. The inclusion criteria were: women between 18 and 60 years old with moderate to severe facial melasma (mMASI > 4), without treatment for at least 45 days, except for the use of sunscreen. Patients with other concomitant facial dermatoses, photosensitive dermatoses, history of hypersensitivity to N-acetylcysteine, and pregnant or lactating women were not included.

The participants were randomized (1:1) comprising 25 blocks with two participants each, based on computer simulation (central randomization), and consecutively allocated according to the numerical sequence of the products packaged in brown envelopes. The NAC group received capsules containing N-acetylcysteine ​​600 mg, taken twice a day for eight weeks, and the PLAC group received placebo capsules, identical in color and shape to the active ingredient. All participants received broad-spectrum (SPF 60) tinted sunscreen and were instructed to apply it every three hours.

The participants were evaluated at the moment of study inclusion (T0) and after eight weeks (T8). The primary outcome was the reduction in the mMASI (modified Melasma Area and Severity Index) score, measured by a researcher blinded to the groups. The secondary outcomes were: MELASQoL (Melasma Quality of Life Scale), colorimetry (Dif-L: Difference between Luminosity *L between healthy skin and melasma), GAIS (Global Aesthetic Improvement Scale) by blinded photographic assessment and adverse effects.

The sample size was calculated to detect a difference greater than 10% in the change in mMASI score between groups at T8, with a correlation coefficient of 0.75 and standard deviation of 18%, considering the power of 80% and an alpha level of 0.05%, resulting in 25 patients in each group (n = 50). Data were analyzed by intention to treat (regarding adherence), and dropouts were not analyzed. Score variations were compared according to the groups and adjusted by the initial values ​​using generalized linear models with robust analysis. GAIS scores were compared using the Mann-Whitney test and p-values <0.05 were considered significant.^7^ The study was approved by the ethics committee and registered with REBEC (RBR-73zrnjh).

Of 64 eligible women, 10 did not meet the inclusion criteria, and four did not agree to participate in the study. Of the 50 included women, 49 completed the study, with one dropout in the PLAC group for a reason unrelated to the treatment. The main clinical and demographic data of the sample are shown in Table 1 and they did not differ between the groups (p > 0.1).

**Table 1:** Main clinical and demographic data of the sample.

	N-Acetylcysteine	Placebo	Total
n	25	25	50
Age (years), mean (sd)	44.1 (7.5)	45.1 (7.4)	44.6 (7.4)
Phototype, n (%)			
III	7 (28%)	7 (28%)	14 (28%)
IV	10 (40%)	11 (44%)	21 (42%)
V	8 (32%)	7 (28%)	15 (30%)
Family history, n (%)	21 (84%)	15 (60%)	36 (72%)
OC, n (%)	6 (24%)	6 (24%)	12 (24%)
Daily sun exposure (min/d), median (p25-p75)	20 (0–60)	20 (0–60)	20 (0–60)
Pregnancies, median (p25-p75)	2 (2–3)	2 (1–3)	2 (1–3)
Age at onset (years), mean (sd)	27.4 (7.5)	30.5 (9.2)	30.0 (8.4)
mMASI, mean (sd)	9.8 (3.7)	10.1 (3.9)	9.8 (3.4)
MELASQoL, mean (sd)	47.7 (15.7)	53.2 (13.7)	50.5 (14.8)
Dif-L, mean (sd)	6.0 (2.3)	5.9 (1.7)	6.0 (2.0)

OC, oral contraceptive; mMASI, modified Melasma Area and Severity Index; MELASQoL, Melasma Quality of Life Scale; Dif-L, Difference in Luminosity (*L) between adjacent skin and melasma.

The outcomes and clinical illustrations are shown in Fig. 1, Fig. 2, Fig. 3. After eight weeks, both groups showed a reduction in the severity score (mMASI): there was a reduction of 12% (95% CI 8 %–19 %) in the NAC group and 12% (95% CI 5%–21%) in the PLAC group (p = 0.613). MELASQoL and colorimetry showed a reduction in participants from both groups (p < 0.03); however, there was no difference in reductions between the groups (p > 0.7). According to photograph analysis, the NAC group resulted in 60% (95% CI 40 %–76 %) of global improvement (GAIS), *versus* 29% (95% CI 12 %–44 %) in the PLAC group (p = 0.317).

**Fig. 1:**
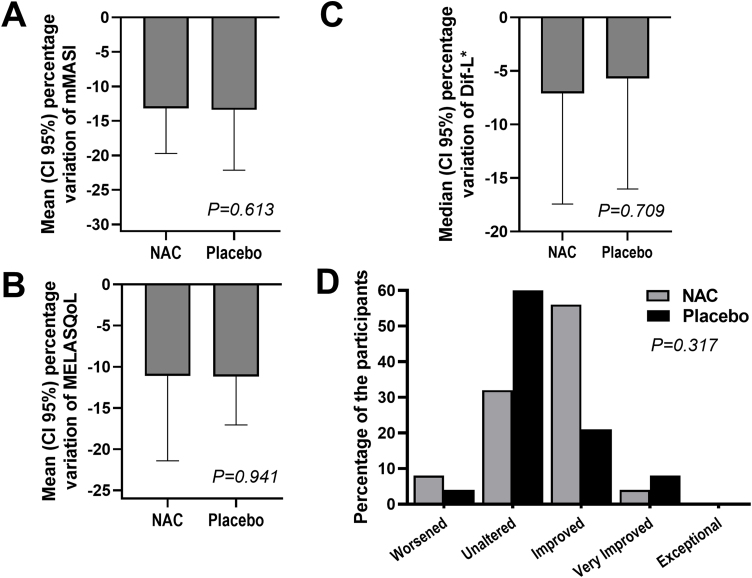
Graphs of the variation in mMASI (A), MELASQoL (B), colorimetry (Dif-*L - C), and assessment of improvement between the studied groups (GAIS ‒ Global Aesthetic Improvement Scale - D), at T8.

**Fig. 2:**
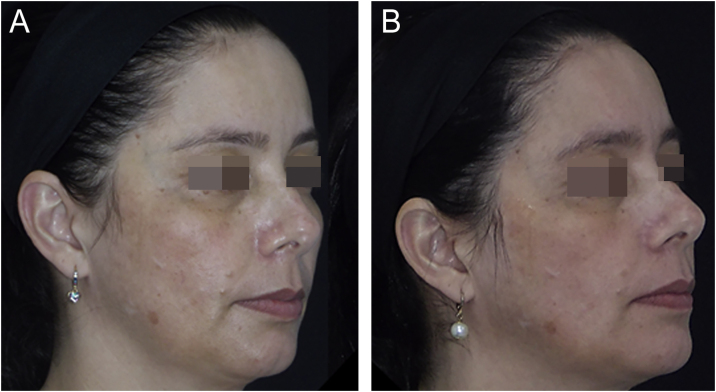
Facial melasma treated with photoprotection and oral N-acetylcysteine for eight weeks. (A) Before treatment. (B) After treatment.

**Fig. 3:**
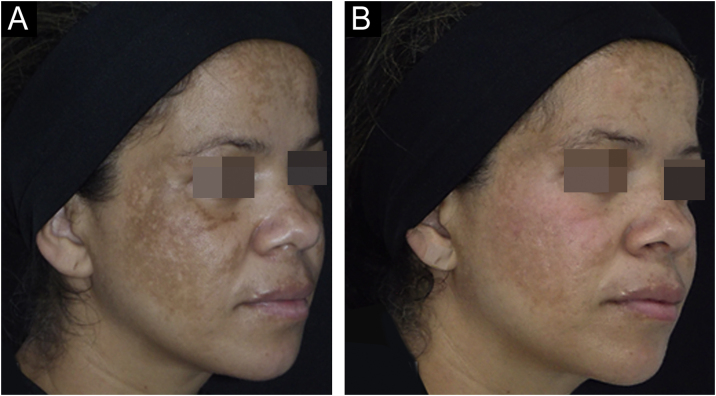
Facial melasma treated with photoprotection and oral placebo for eight weeks. (A) Before treatment. (B) After treatment.

The medication was well tolerated by the participants, and only eight (NAC) and two (PLAC) women reported heartburn or epigastric pain, without the need to interrupt treatment; two (NAC) and three (PLAC) women reported drowsiness, and one woman (NAC) reported a feeling of dry mouth.

This trial was the first to evaluate the use of oral N-acetylcysteine in monotherapy, associated with broad-spectrum sunscreen, for the treatment of melasma. It demonstrated that, although well tolerated, oral N-Acetylcysteine, in the tested regimen, did not reduce the assessed objective and subjective scores, when compared to the performance of the oral placebo.

Oral NAC is a potent antioxidant with the potential to restore intracellular glutathione. In dermatology practice, it is mainly used in the treatment of dermatocompulsions and pseudoporphyria.^8^, ^9^ In a non-randomized trial with 30 Egyptian women, one group (n = 10) received oral glutathione 500 mg/day, while another used topical glutathione 2%, and a third used placebo for four weeks, always associated with SPF 30 sunscreen. Superior performance was observed in the oral and topical groups, with no reports of adverse effects. The study used a small sample, did not employ conventional metrics (e.g., mMASI), and was short in duration; however, their results reiterate the role of antioxidants in the treatment of melasma.^10^

Possible limitations of this study include the brevity of the intervention (eight weeks); however, it is expected that after two cycles of epithelial renewal, minimal interference with melanization can be observed, as evidenced in other therapeutic trials in melasma with oral active ingredients.^6^, ^11^, ^12^ Moreover, the inclusion of only female adults and those with moderate to severe melasma (mMASI > 4) reduces the generalizability of the results. Other studies should be conducted to explore antioxidant substances in the treatment of melasma, or in the prevention of its recurrence.

In conclusion, despite being well tolerated in the used regimen, oral N-acetylcysteine ​​was not effective in the treatment of melasma.

## Financial support

None declared.

## Authors' contributions

Melissa de Almeida Corrêa Alfredo: Design and planning of the study, drafting and editing of the manuscript, and approval of the final version of the manuscript.

Ingrid Rocha Meireles Holanda: Design and planning of the study, drafting and editing of the manuscript, approval of the final version of the manuscript.

Daniel Pinho Cassiano: Design and planning of the study, drafting and editing of the manuscript, and approval of the final version of the manuscript.

Paula Basso Lima: Design and planning of the study, drafting and editing of the manuscript, and approval of the final version of the manuscript.

Ana Cláudia Cavalcante Espósito: Design and planning of the study, drafting and editing of the manuscript, and approval of the final version of the manuscript.

Hélio Amante Miot: Design and planning of the study, drafting and editing of the manuscript, and approval of the final version of the manuscript.

## Conflicts of interest

None declared.

## References

[1] Espósito A.C.C., Cassiano D.P., da Silva C.N., Lima P.B., Dias J.A.F., Hassun K. (2022). Update on melasma-part I: pathogenesis. Dermatol Ther (Heidelb)..

[2] Cassiano D.P., Espósito A.C.C., da Silva C.N., Lima P.B., Dias J.A.F., Hassun K. (2022). Update on melasma-part II: treatment. Dermatol Ther (Heidelb)..

[3] Seçkin H.Y., Kalkan G., Baş Y., Akbaş A., Önder Y., Özyurt H. (2014). Oxidative stress status in patients with melasma. Cutan Ocul Toxicol..

[4] Njoo M.D., Menke H.E., Pavel S., Westerhof W. (1997). N-Acetylcysteine as a bleaching agent in the treatment of melasma. J Eur Acad Dermatol Venereol..

[5] Arjinpathana N., Asawanonda P. (2012). Glutathione as an oral whitening agent: a randomized, double-blind, placebo-controlled study. J Dermatolog Treat..

[6] Lima P.B., Dias J.A.F., Esposito A.C.C., Miot L.D.B., Miot H.A. (2021). French maritime pine bark extract (pycnogenol) in association with triple combination cream for the treatment of facial melasma in women: a double-blind, randomized, placebo-controlled trial. J Eur Acad Dermatol Venereol..

[7] Miola A.C., Miot H.A. (2021). P-value and effect-size in clinical and experimental studies. J Vasc Bras..

[8] Nwankwo C.O., Jafferany M. (2019). N-Acetylcysteine in psychodermatological disorders. Dermatol Ther..

[9] Guiotoku M.M., Pereira Fde P., Miot H.A., Marques M.E. (2011). Pseudoporphyria induced by dialysis treated with oral N-acetylcysteine. An Bras Dermatol..

[10] Farahat A.A., El-Garhy H., El-Mahdy N.A., Ali B.M. (2018). Evaluation of the efficacy and safety of topical and oral glutathione in treatment of melasma. Med J Cairo Univ..

[11] Dias J.A.F., Lima P.B., Cassiano D.P., Esposito A.C.C., Bagatin E., Miot L.D.B. (2022). Oral ketotifen associated with famotidine for the treatment of facial melasma: a randomized, double-blind, placebo-controlled trial. J Eur Acad Dermatol Venereol..

[12] Cassiano D., Esposito A.C.C., Hassun K., Bagatin E., Lima M.M.D.A., Lima E.V.A. (2020). Efficacy and safety of microneedling and oral tranexamic acid in the treatment of facial melasma in women: an open, evaluator-blinded, randomized clinical trial. J Am Acad Dermatol..

